# Intraspecies Variability Affects Heterotypic Biofilms of *Porphyromonas gingivalis* and *Prevotella intermedia*: Evidences of Strain-Dependence Biofilm Modulation by Physical Contact and by Released Soluble Factors

**DOI:** 10.1371/journal.pone.0138687

**Published:** 2015-09-25

**Authors:** Graziela Murta Barbosa, Andrea Vieira Colombo, Paulo Henrique Rodrigues, Maria Regina Lorenzetti Simionato

**Affiliations:** Department of Microbiology, Institute of Biomedical Sciences, University of São Paulo, São Paulo, São Paulo, Brazil; University of Florida, UNITED STATES

## Abstract

It is well known that strain and virulence diversity exist within the population structure of *Porphyromonas gingivalis*. In the present study we investigate intra- and inter-species variability in biofilm formation of *Porphyromonas gingivalis* and partners *Prevotella intermedia* and *Prevotella nigrescens*. All strains tested showed similar hydrophobicity, except for *P*. *gingivalis* W83 which has roughly half of the hydrophobicity of *P*. *gingivalis* ATCC33277. An intraspecies variability in coaggregation of *P*. *gingivalis* with *P*. *intermedia* was also found. The association *P*. *gingivalis* W83/*P*. *intermedia* 17 produced the thickest biofilm and strain 17 was prevalent. In a two-compartment system *P*. *gingivalis* W83 stimulates an increase in biomass of strain 17 and the latter did not stimulate the growth of *P*. *gingivalis* W83. In addition, *P*. *gingivalis* W83 also stimulates the growth of *P*. *intermedia* ATCC25611 although strain W83 was prevalent in the association with *P*. *intermedia* ATCC25611. *P*. *gingivalis* ATCC33277 was prevalent in both associations with *P*. *intermedia* and both strains of *P*. *intermedia* stimulate the growth of *P*. *gingivalis* ATCC33277. FISH images also showed variability in biofilm structure. Thus, the outcome of the association *P*. *gingivalis*/*P*. *intermedia* seems to be strain-dependent, and both soluble factors and physical contact are relevant. The association *P*. *gingivalis*-*P*. *nigrescens* ATCC33563 produced larger biomass than each monotypic biofilm, and *P*. *gingivalis* was favored in consortia, while no differences were found in the two-compartment system. Therefore, in consortia *P*. *gingivalis*-*P*. *nigrescens* physical contact seems to favor *P*. *gingivalis* growth. The intraspecies variability found in our study suggests strain-dependence in ability of microorganisms to recognize molecules in other bacteria which may further elucidate the dysbiosis event during periodontitis development giving additional explanation for periodontal bacteria, such as *P*. *gingivalis* and *P*. *intermedia*, among others, to persist and establish chronic infections in the host.

## Introduction

Oral biofilms consist of interdependent and structured bacterial communities that have heterogeneous architecture with areas of high cell density [1, 2]. Oral bacteria bind to accessible host or bacterial surfaces and form complex communities in an orderly fashion overcoming many environmental challenges, such as salivary flow and low-nutrient conditions. Communities that characterize a healthy oral cavity are composed primarily of commensal bacteria and few are known to be directly responsible for the development of diseases in the mouth. However, certain bacterial species, such as *Porphyromonas gingivalis*, can alter the pathogenic potential of the oral biofilm [3]. Therefore, the presence of certain pathogens and the interactions established between them, other oral bacteria and the host can lead to the development a chronic inflammatory and infectious process in the gum, such as periodontitis [4, 5].

Periodontitis affects supporting tissues of the teeth. It has a multifactorial etiology and is associated with an increase in Gram-negative anaerobes in dental plaque biofilm. Indeed, *P*. *gingivalis*, *Prevotella intermedia* and *Prevotella nigrescens* are frequently isolated from periodontal lesions, but can also be detected in periodontally healthy individuals [6]. These findings could be interpreted as periodontitis is being caused by a broadly-based dysbiotic, synergistic microbiota, where diverse bacteria may be able to fulfill distinct roles that converge to form and stabilize a disease-favoring microbiota. It is likely that in potentially pathogenic communities a variety of bacterial species and/or strains contribute with the genes necessary for these conditions to be satisfied [3]. Several not mutually exclusive explanations may involve variability in the healthy status of the periodontal tissues, but strain and virulence diversity within the population structure of *P*. *gingivalis*, *P*. *intermedia* and *P*. *nigrescens* may affect their ability to act as pathogens [7–13]. Moreover, the interactions of certain strains of keystone and accessory pathogens may be required to disrupt host immune surveillance and elevate the pathogenicity of the periodontal microbiota.

In this report we demonstrate a strain-dependent variability in biofilm formation and structure, particularly for heterotypic biofilms. We show by quantitative PCR (q-PCR) that *P*. *gingivalis* is prevalent in two-species biofilms for all combinations, except one. A two-compartment co-culture system showed soluble factors play a role in biofilm modulation, but also indicated that physical contact is relevant. The contribution of bacteria in periodontal disease progression and their ecological interactions within complex microbial communities, such as the periodontal microbiota, may be influenced through contact-dependent mechanisms, and short range diffusible signals, including metabolic products, all of which can influence the pathogenic potential of the periodontal microbiota [14]. In the periodontal ecosystem diverse strains may be able to interact in different ways either converging or not to form and stabilize a disease-favoring microbiota.

## Material and Methods

### Bacterial strains and culture conditions

*Porphyromonas gingivalis* W83, *Porphyromonas gingivalis* ATCC33277, *Prevotella intermedia* 17, *Prevotella intermedia* ATCC25611 and *Prevotella nigrescens* ATCC33563 were used in this study. All strains were grown in Todd Hewitt medium (Difco) supplemented with yeast extract (5 mg/ml (Difco), menadione (1 μg/ml (Sigma), and hemin (0.5 μg/ml (Sigma) at 37°C in an anaerobic (85% N_2_, 10% H_2_ and 5% CO_2_) chamber (Coy Laboratory Products).

### Hydrophobicity assays

The ability of the strains to adhere to n-hexadecane was used as a measure of their relative surface hydrophobicity as described by [15]. Bacteria were harvested from overnight cultures (O/N– 16 h) grown in supplemented Todd-Hewitt broth (Difco). The organisms were washed twice and suspended in PUM buffer (100 mM K_2_HPO_4_; 50 mM KH_2_PO_4_; 30 mM urea and 10 mM MgSO_4_, pH 7.1). The suspensions were adjusted to an optical density (OD_600_) of 1.0 (BioPhotometer, Eppendorf, Hamburgo-Germany). Quintuplicate samples (3.0 ml) of the bacterial suspensions were placed in glass tubes and 400 μl of n-hexadecane (Sigma) was added and were incubated in a water bath at 30°C for 10 minutes. Each tube was then mixed twice on a Vortex mixer for 30 sec with 5 sec resting period. After 15 min at room temperature (RT) OD_600_ of aqueous phase was determined. The percent of hydrophobicity was calculated as follows:

$$\text{\% of Hydrophobicity }=\;\left[{\text{OD}}_{\text{600}}\text{before mixing – }\left({\text{OD}}_{\text{600}}{\text{after mixing/OD}}_{\text{600}}\text{before mixing}\right)\right]\text{ X 100}$$

The values were expressed as the percentage of bacteria adsorbed into the n-hexadecane phase compared with suspensions without n-hexadecane (control).

### Coaggregation assays

Assays of coaggregation among strains were performed according to [16]. Overnight cultures were harvested by centrifugation (3,000x*g* for 5 min at RT) and washed once with coaggregation buffer (1.0 mM Tris buffer pH 8.0; 0.1 mM CaCl_2_; 0.1 mM MgCl_2_; 150 mM NaCl). All bacterial suspensions were adjusted to OD_600_ = 1.0. Glass tubes containing two species were prepared by mixing 1.5 ml portions of appropriate combinations of *P*. *gingivalis*, *P*. *intermedia* and *P*. *nigrescens*, and controls were prepared with 3.0 ml of one species suspensions. All glass tubes were kept at 37°C under aerobic conditions. Every 60 min, over a period of 6 h, 500 μl of the upper phase of bacterial solutions was transferred into an acrylic cuvette and OD_600_ was recorded. The coaggregation degree was calculated as follows, where OD_A_ and OD_B_ stand for the optical densities of bacteria A and B, and OD_A + B_ corresponds the OD of the mixture of bacteria A and B after 6hrs of contact.

$$\text{\%Coaggregation }=\;\left\{\left[\left({\text{OD}}_{\text{A}}{+\text{OD}}_{\text{B}}\right)\text{ – }\left({\text{2 X OD}}_{\text{A+B}}\right)\right]\text{/}\left({\text{OD}}_{\text{A}}{+\text{ OD}}_{\text{B}}\right)\right\}\text{ x 100}$$

**Fig 1:**
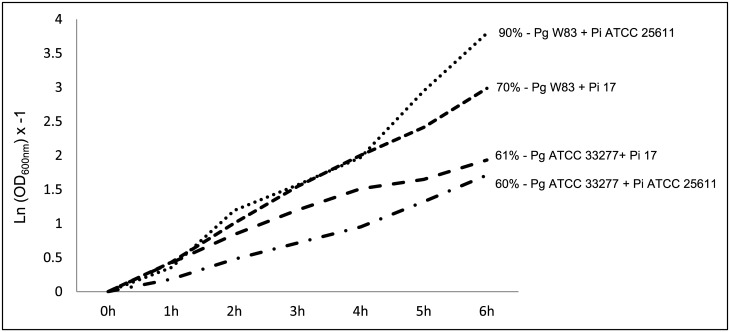
Coaggregation profiles of *Porphyromonas gingivalis* W83 and ATCC33277, *Prevotella intermedia* 17 and ATCC25611. Percentage numbers showed in the end of each line represents the coaggregation percentage at 6 h of incubation for each bacterial association.

In order to provide a better understanding of the coaggregation along the incubation period in Fig 1 data were presented as the natural logarithm of OD_600_ at each time period X [–1].

### Quantification of biofilm biomass

Homotypic and heterotypic biofilms were quantified by a microtiter plate assay according to [17]. Briefly, overnight cultures were diluted to OD_600_ = 0.5 and let grown up to OD_600_ = 1.0 at 37°C under anaerobic conditions. Aliquots of the cultures (one-species biofilm—500 μl; two-species biofilm—250 μl of each) were then distributed into 48 wells flat-bottom (Jet Biofil, China) containing 9 mm diameter glass coverslips (Knittel Glass, Germany) and incubated for 24 h at 37°C under anaerobic conditions. Biofilms were then washed once with 500 μl of buffer PBS, stained with 0.4% safranin for 15 min, washed three times by immersion in distilled water and distained with 95% ethanol for 15 min. The extracted dye was then quantified by measuring absorbance at 490 nm with a microplate reader (Model 680; Bio-Rad Laboratories). Biofilm assays were repeated independently three times and in quintuplicate. Results were expressed as mean ± standard deviation.

### Quantification of biofilm biomass in a two-compartment separated co-culture system

Assays of biofilm formation into 24 well-plate co-culture system of two compartments separated by a 0.4 μm pore size membrane (BD Falcon) were performed as described by [16]. Bacterial strains were cultured as previously described (Quantification of biofilm biomass). Upon cultures reaching OD_600_ = 1.0, 500 μl of each bacterial culture were inoculated into the lower compartment and another 500 μl of its co-culture partner were inoculated into the upper compartment. The plates were then incubated for 24 h at 37°C under anaerobic conditions. After 24 h, the membrane inserts were removed and the biomass in the lower compartment was determined as previously described (Quantification of biofilm biomass). Controls consisted of same strain inoculated in both compartments. The assays were repeated independently three times and in triplicate.

### DNA extraction and quantification

DNA was extracted from 24 h heterotypic biofilms cultured into 24 flat-bottom well-plate coated with 13 mm diameter glass coverslips by WIZARD Genomic DNA Purification kit (Promega), according to the manufacturer’s instructions. Prior to DNA extraction, biofilms were washed once with PBS to remove non-attached bacteria. Genomic DNA was stored at -20°C.

### q-PCR quantification of strains in heterotypic biofilms

Quantification of *P*. *gingivalis*, *P*. *intermedia* and *P*. *nigrescens* in biofilms was performed by q-PCR analysis using the SYBR green dye to detect the species-specific amplicons of the single copy gene *waaA* [18]. Primers for conservative region of the single copy *thiC* were designed with the Vector NTI program (Life Technologies) and used as internal control (Table 1).

**Table 1:** Primer sequences used for quantitative real-time PCR (q-PCR).

Target genes	Primers (5’– 3’)	Product size (bp)	Locus tag (NIH)
*P*. *gingivalis waaA* ^1^	F: TGGTTTCATGCAGCTTCTTTR: TCGGCACCTTCGTAATTCTT	146	PGN_RS02590
*P*. *intermedia waaA* ^1^	F:GACCCGAACGCAAAATACATR:AGGGCGAAAAGAACGTTAGG	130	PIN17_RS06380
*P*. *nigrescens waaA* ^*2*^	F: GGCTGCTGGACACTCCAAGGCTTTR:GCTATGATGAGTTTCCACTCGCTGTG	120	ZP 08672979
*P*. *gingivalis thiC* ^*2*^	F: ACGAYGCCAAYGATGCTGCR: ATCTTGTGCATBGGYACGTGTCC	124	PGN_RS00750
*P*. *intermedia thiC* ^*2*^			PIN17_RS04875
*P*. *nigrescens thiC* ^*2*^			ZP 08673195

Source:

^1^ Hyvarinen et al., 2009;

^2^ This study

Each reaction mixture contained 2.0 μl of template DNA (10 ng/μl), 10 μl of Fast Plus EvaGreen Mastermix (Biotium), 1.0 μl each of the appropriate forward and reverse primers (final concentration, 200 nM each) and 6.0 μl of sterilized nuclease-free water (Ambion, Life Technologies). Amplification reactions were performed in a thermocycler iCycler (Bio-Rad Laboratories) using optical-grade 96-well plate with the following thermal cycle: 95°C for 5 min followed by 40 cycles at 95°C for 15 sec and 60°C for 60 sec. During the annealing and extension steps, fluorescence emissions were monitored and data were collected by iCycler iQ Software (Bio-Rad Laboratories). Non-template controls were included for all reactions. Dissociation curves were generated by incubating amplification products at 60°C with successive increase in temperature of 0.4°C for 10 sec up to 95°C. Analysis of the melting curves with all primer sets revealed a single sharp peak. The purity of the amplified product was checked by comparing its dissociation curve with the dissociation curve of each respectively purified PCR product.

The internal control was used to normalize the *waaA* copy number by dividing the *waaA P*. *gingivalis* or *P*. *intermedia* or *P*. *nigrescens* copy numbers of each sample by its corresponding *thiC* copy number. The relative quantification of each species in heterotypic biofilms was calculated by LinRegPCR (11.0) software [19], which takes into account the amplification efficiency of each reaction. The data presented are the mean of three independent biofilms.

### Fluorescence *in situ* hybridization (FISH)

FISH protocol used was described by [20]. Oligonucleotide probes designed for the 16S rRNA gene region [21] were used to visualize *P*. *gingivalis* and *P*. *intermedia* (Table 2).

**Table 2:** Oligonucleotide probes used for FISH assays.

Probe^◆^	Primer/probe sequence (5'-3')	Target
**POGI** *	CAATACTCGTATCGCCCGTTATTC	*P*. *gingivalis*
**PRIN** **	CTTTACTCCCCAACAAAAGCAGTTTACAA	*P*. *intermedia*

* labeled with CY5 (Sulfoindocyanine dye—Bioneer);

** labeled with FITC (Fluorescein isothiocyanate dye—Molecular Probes); Source: Sunde et al., 2003.

Biofilms were developed on chamber glass slides (CultureSlides—Corning) as previously described. After 24 h of incubation, the planktonic fraction was aspirated and biofilms were fixed with 500 μl of 3.7% formaldehyde solution for 12 h at 4°C. The bacterial cells were then permeabilized with 200 μl Triton X-100 0.5% (BioBasic) at 37°C for 10 min in a humid chamber and washed once with nuclease free water. One hundred microliters of freshly prepared hybridization solution (50 ng/ml of each probe in 20 mM Tris-HCl pH 7.6; 0.9 mM NaCl; 0.01% SDS and 30% of formamide) was added onto the fixed biofilms and slides were then incubated for 2 h in a dark-humid chamber at 46°C. Slides were washed once with 200 μl of wash solution (20 mM Tris-HCl pH 7.6; 0.9 mM NaCl; 5 mM EDTA and 0.01% of SDS) for 15 min at 50°C, rinsed with 200 μl of distilled water and air dried in the dark at room temperature. One aliquot of 10 μl of ProLong Gold anti-fade reagent (Molecular Probes) was added to the glass slides which were then sealed with coverslips.

### Confocal microscopy

A laser-scanning confocal microscope (Axiovert, 100M/LSM 510 Carl Zeiss, Oberkochen, Germany) was used to record confocal image in random locations, after which biofilm thicknesses were determined by ZEISS LSM Image Browser software (3.5.0.223, Carl Zeiss). Sections of confocal scanning were scanned with increments of stacks (ranging from 0.5–1.0 μm) using an excitation wavelength of 488 and 543 nm. Biofilm thicknesses were determined by selecting the midpoint of the slices and mean thickness and standard deviation were calculated.

### Statistical Analysis

All data were obtained from three independent experiments and each condition was included at least in triplicate in each experiment. Sample distributions were checked using ANOVA and nonparametric Kruskal-Wallis test followed by nonparametric post-tests to determine the statistical significance (Statgraphics, Centurion XVI). A significance level of *p* < 0.05 was considered significant.

## Results

### Hydrophobicity assays

All bacteria tested showed similar hydrophobicity, except *P*. *gingivalis* W83 which has roughly half of the hydrophobicity of *P*. *gingivalis* ATCC33277 after 10 min of suspension in n-hexadecane buffer (Table 3)

**Table 3:** Hydrophobicity of strains of *P*. *gingivalis*, *P*. *intermedia* and *P*. *nigrescens*.

Type Strains	% Bacteria in the n-hexadecane phase
*P*. *gingivalis* W83	22.9
*P*. *gingivalis* ATCC33277	46.7
*P*. *intermedia* 17	51.9
*P*. *intermedia* ATCC25611	51.2
*P*. *nigrescens* ATCC33563	48.4

### Coaggregation assays

*P*. *gingivalis* showed remarkably different coaggregation percentages when mixed with the two strains of *P*. *intermedia* (Fig 1). The highest coaggregation percentage was determined for *P*. *gingivalis* W83 mixed with *P*. *intermedia* ATCC25611, which was 20% higher than *P*. *gingivalis* W83 mixed with *P*. *intermedia* 17 at 6 h. Interestingly, up to 4 h of coaggregation both strains had comparable coaggregation percentages with W83. *P*. *gingivalis* ATCC33277 had similar coaggregation percentages with both strains of *P*. *intermedia* at 6 h, but not at earlier time points. These results indicate an intraspecies variability in the coaggregation of *P*. *gingivalis* with *P*. *intermedia*. *P*. *nigrescens* ability to coaggregate with strains of *P*. *gingivalis* and *P*. *intermedia* were virtually null (data not shown).

### Quantification of biofilm biomass

**Fig 2:**
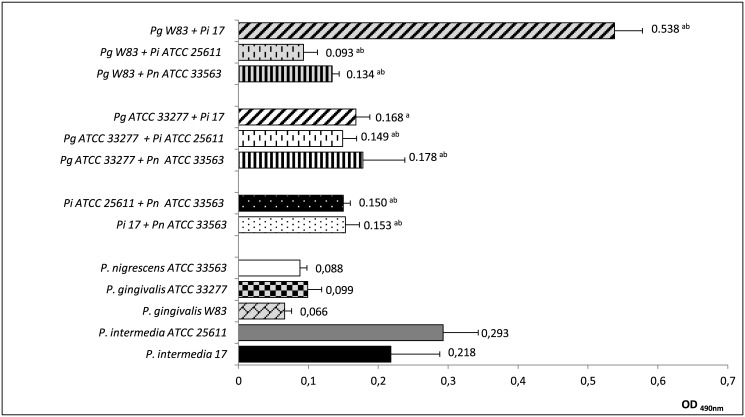
Quantification of biomass (OD_490_) in two-species biofilms of *Porphyromonas gingivalis*, *Prevotella intermedia* and *Prevotella nigrescens*. ^**a**^ means significant difference between values of heterotypic biofilm and values of monotypic biofilm of the first strain in the association (Kruskal-Wallis test followed by nonparametric post-tests, *p* < 0.01); ^**b**^ means significant difference between values of heterotypic biofilm and values of monotypic biofilm of the second strain in the association (Kruskal-Wallis test followed by nonparametric post-tests, *p* < 0.01).

Enhanced growth for both *P*. *gingivalis* strains in two-species biofilms was observed when *P*. *intermedia* was present as one of the partners. But, different behavior was observed among strains (Fig 2). *P*. *intermedia* ATCC25611 produced a larger biomass in monotypic biofilm than in association with either strain of *P*. *gingivalis*. The biomass of the association *P*. *gingivalis* ATCC33277/*P*. *intermedia* 17 was not statistically significantly different than *P*. *intermedia* 17 monotypic biomass. However, the association *P*. *gingivalis* W83/*P*. *intermedia* 17 produced an increased biomass than either monotypic biofilm. The biovolumes of these two species increased two and half- and eight-fold, respectively. These results indicate the association *P*. *gingivalis*/*P*. *intermedia* seems to be beneficial for *P*. *gingivalis* and detrimental for *P*. *intermedia*.

The biomass in two-species biofilm increased when *P*. *nigrescens* ATCC33563 was present as one of the partners of each *P*. *gingivalis* strain (Fig 2). The data obtained show there was mutualistic growth for the pair *P*. *gingivalis*/*P*. *nigrescens*. However, the biomass of the heterotypic biofilm has decreased when *P*. *nigrescens* was co-cultivated with either strain of *P*. *intermedia* when compared to the biomass of each *P*. *intermedia* monotypic biofilm. Point of worthy, the overall biomass of the *P*. *intermedia*/*P*. *nigrescens* combinations was larger than the biomass of *P*. *nigrescens* monotypic biofilm.

### Quantification of biofilm biomass in a two-compartment co-culture system

**Fig 3:**
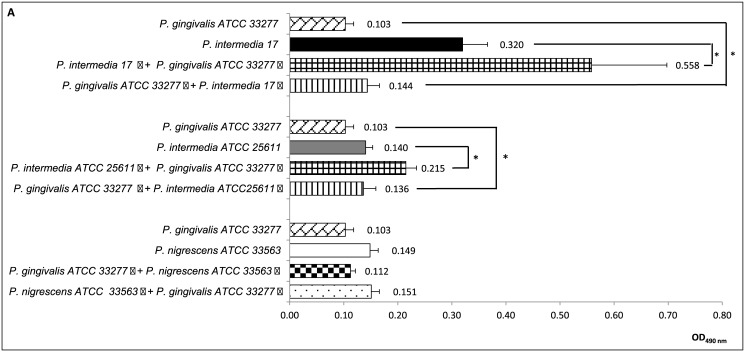
Effect of co-culture of *Porphyromonas gingivalis*, *Prevotella intermedia* and *Prevotella nigrescens* on biofilm formation in a two-compartment co-culture system. **Panel A**: *P*. *gingivalis* ATCC33277 and co-culture partners *P*. *intermedia* strains 17 and ATCC25611, and *P*. *nigrescens* ATCC33563. **Panel B**: *P*. *gingivalis* W83 and co-culture partners *P*. *intermedia* strains 17 and ATCC25611, and *P*. *nigrescens* ATCC33563. **Panel C**: *P*. *intermedia* strains 17 and ATCC25611 and co-culture partner *P*. *nigrescens* ATCC33563 Values represent average of 3 experiments (Kruskal-Wallis test followed by nonparametric post-tests, *p < 0.01). **a** denotes strain in the lower compartment (biomass measured) and **b** denotes strain in the upper compartment.

An enhanced growth of *P*. *gingivalis* ATCC33277 biofilm was observed in a two-compartment co-culture separated by a porous membrane when either strain of *P*. *intermedia* was present as one of the partners while the biomass of *P*. *gingivalis* W83 was not affected. In addition, both strains of *P*. *gingivalis* stimulated the growth of *P*. *intermedia*. However, no effects were observed in a *P*. *gingivalis*/*P*. *nigrescens* co-culture system. Furthermore, *P*. *nigrescens* stimulates the growth of *P*. *intermedia* 17 biofilm while the biomass of strain ATCC26511 was not affected. (Fig 3A, 3B and 3C). These findings indicate soluble factors produced by *P*. *gingivalis* increase biofilm formation of *P*. *intermedia*. Moreover, *P*. *intermedia* also produces soluble factors that enhances biofilm formation of *P*. *gingivalis*, but only for strain ATCC33277, which may indicate strain-dependent biofilm-stimulus substances. Similar findings were determined for *P*. *nigrescens* which stimulates the biofilm growth of *P intermedia* 17 but not *P*. *intermedia* ATCC26511 strain.

### q-PCR of heterotypic biofilms

**Fig 4:**
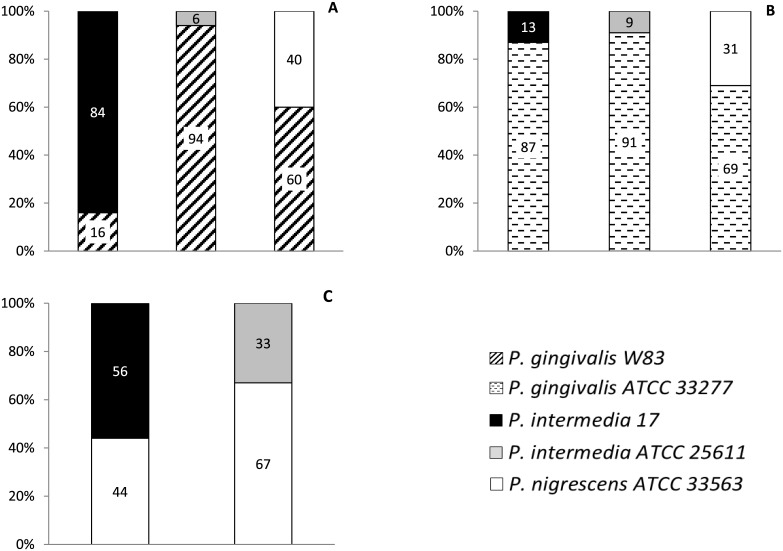
Values inside the bars represent the percentage (%) of each strain in two-species biofilm measured by q-PCR after 24 hrs of incubation. **Panel A**: two-species biofilms of *P*. *gingivalis* W83 mixed with either *P*. *intermedia* 17, *P*. *intermedia* ATCC25611 or *P*. *nigrescens* ATCC33563; **Panel B**: two-species biofilms of *P*. *gingivalis* ATCC33277 mixed with either *P*. *intermedia* 17, *P*. *intermedia* ATCC25611 or *P*. *nigrescens* ATCC 33563; **Panel C**: two-species biofilms of *P*. *nigrescens* ATCC33563 mixed with either *P*. *intermedia* 17 or *P*. *intermedia* ATCC25611.

Since the data obtained by biomass quantification may indicate a contact-dependent modulation in biofilm formation, we have determined the amount of each species in two-species biofilms by using species-specific primers and performed q-PCR (Fig 4A, 4B and 4C). In *P*. *gingivalis*-*P*. *intermedia* consortia, *P*. *gingivalis* was prevalent, ranging 87–94% of the total biomass. However, in the *P*. *gingivalis* W83-*P*. *intermedia* 17 consortium the majority of the cells present in the biofilm was *P*. *intermedia* 17 (84%). In biofilms containing *P*. *gingivalis*-*P*. *nigrescens*, *P*. *gingivalis* was prevalent, but in *P*. *intermedia*-*P*. *nigrescens* biofilms, a distinct strain-dependent behavior was observed. *P*. *nigrescens* was prevalent in association with *P*. *intermedia* ATCC25611, but not prevalent in association with strain 17. These data show that *P*. *gingivalis* growing in contact with *P*. *intermedia* and *P*. *nigrescens* is the most established bacterial species in these associations. Nevertheless, this phenomenon is strain-dependent.

### FISH and confocal microscopy

**Fig 5:**
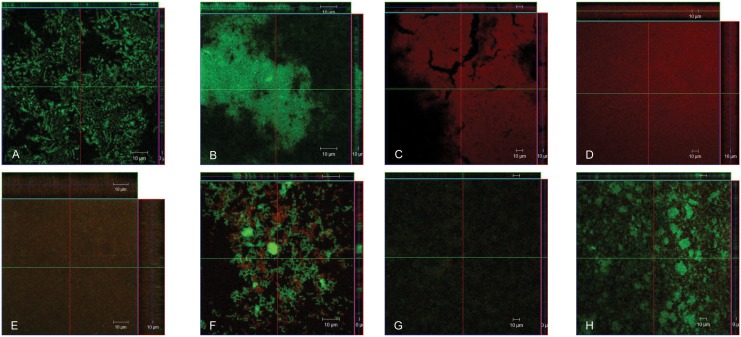
Representative confocal micrographs of 24 hrs biofilms of *P*. *gingivalis* and *P*. *intermedia* in chamber cells and thickness measurements. **Panel A**: *P*. *gingivalis* W83 (100x); **Panel B**: *P*. *gingivalis* ATCC33277 (100x); **Panel C**: *P*. *intermedia* 17 (40x); **Panel D**: *P*. *intermedia* ATCC25611 (40x); **Panel E**: *P*. *gingivalis* W83 + *P*. *intermedia* 17 (100x); **Panel F**: *P*. *gingivalis* ATCC33277 + *P*. *intermedia* 17 (100x); **Panel G**: *P*. *gingivalis* W83 + *P*. *intermedia* ATCC25611 (40x); **Panel H**: *P*. *gingivalis* ATCC33277 + *P*. *intermedia* ATCC25611 (40x). Bacterial cells were stained with species-specific fluorophore-conjugated probes.

*P*. *gingivalis* and *P*. *intermedia* monocultures and associations were inoculated into chamber cells for 24 h and images of the biofilms formed were then obtained. Their bio-thickness was also quantified (Table 4). Among the homotypic biofilms, *P*. *gingivalis* W83 showed the scarcest arrangement and thinnest layer (Fig 5A). The consortium *P*. *gingivalis* W83-*P*. *intermedia* 17 was the thickest biofilm (Fig 5E) while *P*. *gingivalis* W83-*P*. *intermedia* ATCC25611 was the thinnest (Fig 5G). In addition, distinct biofilm structures could be observed. Biofilms of *P*. *gingivalis* ATCC33277 in association with strain 17 (Fig 5F) or strain ATCC 25611 (Fig 5H) showed a microcolony arrangement while biofilms of *P*. *gingivalis* W83 in association with either strain of *P*. *intermedia* showed a homogeneous arrangement (Fig 5E and 5G). These findings are consistent with biomass quantification data and strengths the strain-dependent modulation of biofilm formation of *P*. *gingivalis* with *P*. *intermedia*.

**Table 4:** Thickness of monotypic and heterotypic biofilms (μm) of *Porphyromonas gingivalis* and *Prevotella intermedia*.

Strains	Average ± sd (μm) (n = 3)
*P*. *gingivalis* W83	2.45 ± 0.2
*P*. *gingivalis* ATCC 33277	4.0 ± 1.7
*P*. *intermedia* 17	12.2 ± 0.4
*P*. *intermedia* ATCC 25611	16.5 ± 0.2
*P*. *gingivalis* W83 + *P*. *intermedia* 17	17.84 ± 0.3
*P*. *gingivalis* ATCC 33277 + *P*. *intermedia* 17	4.54 ± 0.3
*P*. *gingivalis* W83 + *P*. *intermedia* ATCC 25611	3.23 ± 0.5
*P*. *gingivalis* ATCC 33277 + *P*. *intermedia* ATCC 25611	5.5 ± 0.1

## Discussion

Biofilm formation occurs through events of competitive survival and cooperative associations among varied species present in the environment. Oral microorganisms have adhesion mechanisms among themselves and to the surfaces of the mouth primarily to overcome the constant flow of saliva and swallowing reflexes that hinder the establishment of a biofilm. In the present study, we investigated intra- and inter-species variability among *P*. *gingivalis*, *P*. *intermedia* and *P*. *nigrescens* when evolving biofilm.

The relative surface hydrophobicity may play a role on adherence. Our findings indicate similar hydrophobic activities among the studied species except for *P*. *gingivalis* W83. Naito et al (1993) and Davey & Duncan (2006) have also described that *P*. *gingivalis* W83 has a lower hydrophobicity than *P*. *gingivalis* ATCC33277 [22, 23]. Our study demonstrates *P*. *gingivalis* strain-specificity when co-aggregating with *P*. *intermedia*. A higher percentage of coaggregation was found when *P*. *gingivalis* W83 was interacting with both *P*. *intermedia* strains when compared to strain ATCC33277. In a previous study, a heat-labile and proteinous factor on the cell surface of *P*. *gingivalis*, maybe the gingipain-adhesin complex, was shown to be involved in different coaggregation patterns of various strains of *P*. *gingivalis* and *P*. *intermedia* [24].

Although the recognition between the partners is considered an important step to the establishment of bacteria in biofilm, the ecological interactions that take place within the biofilm formation may overcome the initial recognition. In monotypic biofilm *P*. *gingivalis* and *P*. *nigrescens* strains produced thinner biofilms while both strains of *P*. *intermedia* produced thicker biofilms. FISH/Confocal microscopy images showed that the association *P*. *gingivalis* W83-*P*. *intermedia* 17 produced the thickest biofilm. The increase was 8-fold and 2.5-fold higher than homotypic biofilm of *P*. *gingivalis* W83 and *P*. *intermedia* 17, respectively, which suggests a synergistic relation between the strains. The q-PCR results showed strain 17 was prevalent which indicates this association is beneficial for the latter strain. In addition, the presence of strain W83 in the upper well in a two-compartment system improved strain 17 biomass. However, biomass of W83 did not increase when strain 17 was co-cultured in the upper well suggesting that soluble factors released by *P*. *gingivalis* W83 may play a role in the synergistic effect observed. Conversely, FISH and biomass data showed that the association of *P*. *gingivalis* W83-*P*. *intermedia* ATCC25611 produced the thinnest mixed biofilm, although an increase was observed when compared to *P*. *gingivalis* W83 monotypic biofilm. Meanwhile, almost a three times reduction was determined when compared to values of *P*. *intermedia* ATCC25611 monotypic biofilm. In addition, q-PCR data demonstrated that strain W83 was prevalent, but soluble factors do not explain the enhanced prevalence of W83 in the heterotypic biofilm. In this association physical contact may play a role for the benefit of *P*. *gingivalis* W83. In biofilms of *P*. *gingivalis* ATCC33277 with both strains of *P*. *intermedia* there was an increase in heterotypic biomass compared to monotypic biofilm of *P*. *gingivalis* ATCC33277 but a decrease when comparing with *P*. *intermedia* ATCC25611 monotypic biofilm. Also, confocal microscopy showed increased biofilm thickness with both strains of *P*. *intermedia*. In addition, q-PCR data showed that *P*. *gingivalis* ATCC33277 was prevalent in both heterotypic biofilms, and in a two-compartment system the growth of *P*. *gingivalis* ATCC33277 was stimulated by both strains of *P*. *intermedia*. Nevertheless, an enhanced growth for both strains of *P*. *intermedia* was observed in two-compartment co-culture when either strain of *P*. *gingivalis* was present as one of the partners in the upper well compartment. The overall data have shown that *P*. *intermedia* enhances the growth of *P*. *gingivalis* but this event is strain-dependent, and physical contact may be important for the outcome of the heterotypic biofilm.

The association *P*. *gingivalis*-*P*. *nigrescens* ATCC33563 produced larger biomass than each monotypic biofilm suggesting a benefit for both species in the mixed biofilm. However, q-PCR data showed *P*. *gingivalis* was favored in consortia with *P*. *nigrescens*, while no differences were found in the two-compartment system. Thus, in consortia *P*. *gingivalis*-*P*. *nigrescens* physical contact seems to favor *P*. *gingivalis* growth. On the other hand, in mixed biofilms of *P*. *nigrescens* ATCC33563-*P*. *intermedia* biomasses were larger than monotypic biofilm of *P*. *nigrescens* ATCC33563 but smaller than each monotypic biofilm of *P*. *intermedia*, suggesting a favorable relation to *P*. *nigrescens*, confirmed by q-PCR data showing *P*. *nigrescens* was prevalent when associated with *P*. *intermedia* ATCC25611. Our findings in the two-compartment system showed neither *P*. *nigrescens* nor *P*. *intermedia* ATCC25611 stimulate the growth of each other. But, when *P*. *nigrescens* was in association with *P*. *intermedia* 17 the latter was prevalent. Two-compartment system data showed *P*. *nigrescens* stimulates the growth of *P*. *intermedia* 17 while it was not stimulated by strain 17. Overall, the outcome of *P*. *intermedia*-*P*. *nigrescens* consortia is strain-dependent, and both physical contact and soluble factors may be relevant.

In this present study, although the species have been mixed in identical proportions for analysis of biofilm formation this profile was not maintained. This could be due to the drop of homeostatic balance between the strains, resulting in prevalence of certain strains in the biofilm and depends on the strain associated. *P*. *gingivalis* was prevalent in heterotypic biofilm in all performed associations, except in association with *P*. *intermedia* 17. Synergistic and competitive interactions among bacterial strains could explain the phenomenon observed. Using a trans-well system and q-PCR, we have shown either diffusible molecules or physical contact can modify the overall association and the biofilm structure. The FISH technique and confocal microscopy analysis provide measurement of the thickness of biofilms and visual inspection of the structural conformation. Our findings showed an evenly, but also randomly, distribution of the strains provoking changes in the biofilm framework. *In vivo* observations of oral biofilms showed that *P*. *gingivalis*, *P*. *intermedia* or *P*. *nigrescens* were evenly distributed in the top and intermediate layer of the biofilm [25]. However, other study showed *P*. *gingivalis* and *P*. *intermedia* were found in distinct microcolonies in subgingival biofilm [26]. The opposite findings in the distribution of these pathogens in subgingival biofilm region may be due to interactions established between different strains. Also, the release of metabolic products and by-products by different strains of these pathogens, may modulate the biofilm independently of the cell-cell contact. The heterotypic biofilms are modulated not only quantitatively but also qualitatively. Thereby, oral biofilm is not the mere sum of its constituent pathogens and their persistence occurs by the diversity found in the population structure of each pathogen [8, 9] and the inter-relations between them. Nevertheless, the virulence features of varied strains of *P*. *gingivalis* maybe expressed in the context of a heterotypic microbial community [3, 27].

The intraspecies variability found in our study suggests strain-dependence in ability of microorganisms to recognize molecules in other bacteria. *P*. *gingivalis* is one of the primary agents responsible for periodontitis and its diverse population structure [9] interacting with different strains of other microorganisms can elucidate the role of oral pathobionts in dysbiosis during periodontitis development [3, 28, 29]. Therefore, ecological relationships established into the biofilm could confer metabolic advantages for the associated strains giving explanation for the ability of periodontal bacteria, such as *P*. *gingivalis* and *P*. *intermedia*, among others, to persist and establish chronic infections in the host. Several mechanisms by which such bacteria contribute to inflammation and disease have remained hidden and could be important for treating inflammatory diseases of polymicrobial etiology like periodontitis. Further investigations down to the molecular level and *in vivo* studies should be pursued in order to understand the mechanisms involved in the consortium of oral pathogens, underlining the population structure of its constituents, to remodel a normally symbiotic microbiota into a dysbiotic disease-favoring microbiota may provide new therapeutic approaches for periodontitis.
