# Correction: Intraspecies Variability Affects Heterotypic Biofilms of *Porphyromonas gingivalis* and *Prevotella intermedia*: Evidences of Strain-Dependence Biofilm Modulation by Physical Contact and by Released Soluble Factors

**DOI:** 10.1371/journal.pone.0143903

**Published:** 2015-11-25

**Authors:** Graziela Murta Barbosa, Andrea Vieira Colombo, Paulo Henrique Rodrigues, Maria Regina Lorenzetti Simionato

The image for Fig 3 is missing Fig 3B and 3C. Please see the corrected Fig 3 here.

**Fig 3:**
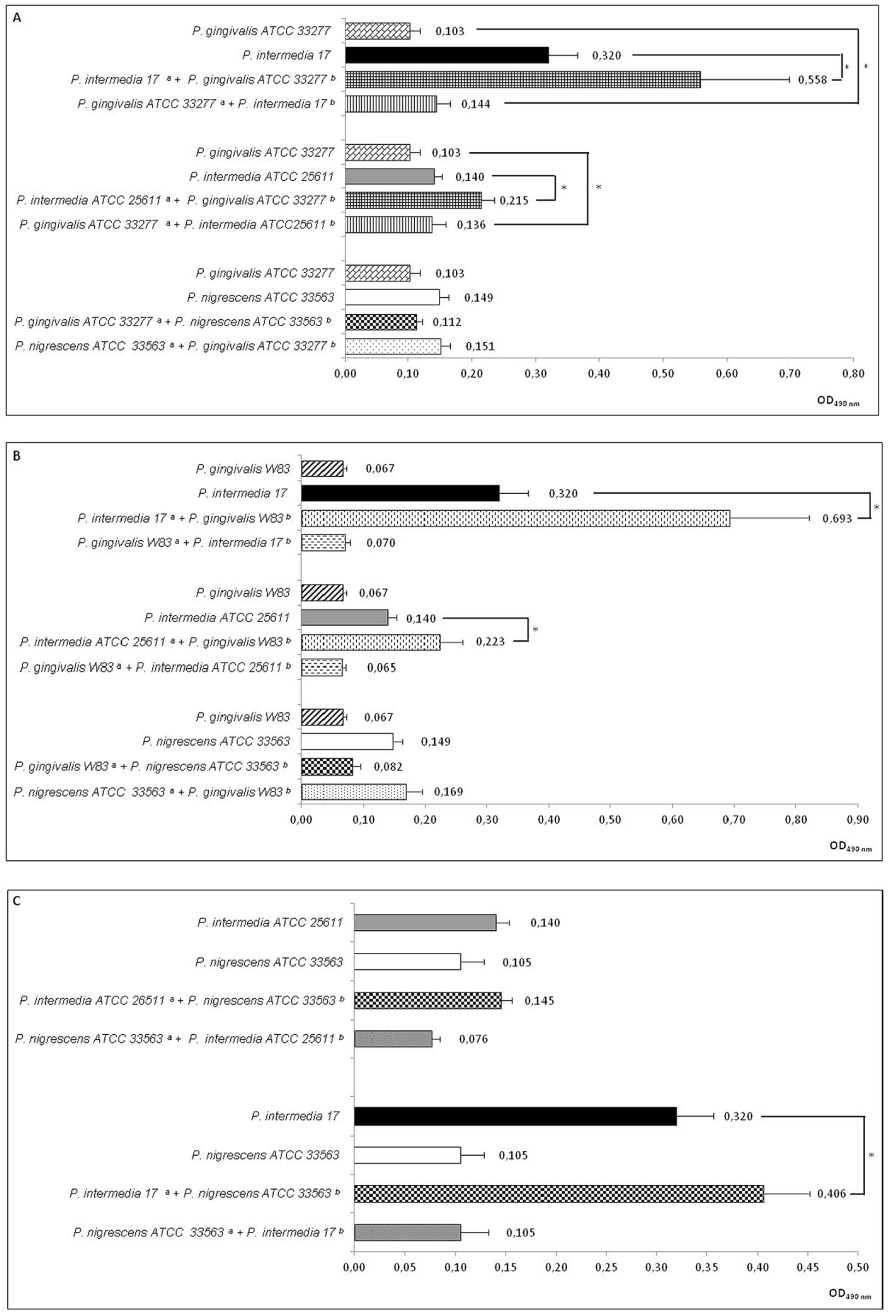
Effect of co-culture of Porphyromonas gingivalis, Prevotella intermedia and Prevotella nigrescens on biofilm formation in a two-compartment co-culture system. **Panel A**: P. gingivalis ATCC33277 and co-culture partners P. intermedia strains 17 and ATCC25611, and P. nigrescens ATCC33563. **Panel B**: P. gingivalis W83 and co-culture partners P. intermedia strains 17 and ATCC25611, and P. nigrescens ATCC33563. **Panel C**: P. intermedia strains 17 and ATCC25611 and co-culture partner P. nigrescens ATCC33563 Values represent average of 3 experiments (Kruskal-Wallis test followed by nonparametric post-tests, *p < 0.01). **a** denotes strain in the lower compartment (biomass measured) and **b** denotes strain in the upper compartment.

Table 1 has been corrected for improved readability. Please see the corrected Table 1 here.

**Table 1:** Primer sequences used for quantitative real-time PCR (q-PCR).

Target genes	Primers (5’– 3’)	Product size (bp)	Locus tag (NIH)
*P*. *gingivalis waaA* ^1^	F: TGGTTTCATGCAGCTTCTTT	146	PGN_RS02590
	R: TCGGCACCTTCGTAATTCTT		
*P*. *intermedia waaA* ^1^	F:GACCCGAACGCAAAATACAT	130	PIN17_RS06380
	R:AGGGCGAAAAGAACGTTAGG		
*P*. *nigrescens waaA* ^2^	F: GCTGCTGGACACTCCAAGGCTTT	120	ZP 08672979
	R: GCTATGATGAGTTTCCACTCGCTGTG		
*P*. *gingivalis thiC* ^2^	F: ACGAYGCCAAYGATGCTGC		PGN_RS00750
*P*. *intermedia thiC* ^2^	R: ATCTTGTGCATBGGYACGTGTCC	124	PIN17_RS04875
*P*. *nigrescens thiC* ^2^			ZP 08673195

Source:

^1^ Hyvarinen et al., 2009;

^2^ This study
